# A genealogical map of the concept of *habit*

**DOI:** 10.3389/fnhum.2014.00522

**Published:** 2014-07-21

**Authors:** Xabier E. Barandiaran, Ezequiel A. Di Paolo

**Affiliations:** ^1^Department of Philosophy, University School of Social Work, UPV/EHU University of the Basque CountryVitoria-Gasteiz, Spain; ^2^Department of Logic and Philosophy of Science, IAS-Research Center for Life, Mind, and Society, UPV/EHU University of the Basque CountryDonostia - San Sebastián, Spain; ^3^Ikerbasque, Basque Foundation for ScienceBilbao, Spain; ^4^Department of Informatics, Centre for Computational Neuroscience and Robotics, University of SussexBrighton, UK

**Keywords:** habit, associationism, organicism, history of psychology, history of philosophy

## Abstract

The notion of information processing has dominated the study of the mind for over six decades. However, before the advent of cognitivism, one of the most prominent theoretical ideas was that of *Habit*. This is a concept with a rich and complex history, which is again starting to awaken interest, following recent embodied, enactive critiques of computationalist frameworks. We offer here a very brief history of the concept of habit in the form of a genealogical network-map. This serves to provide an overview of the richness of this notion and as a guide for further re-appraisal. We identify 77 thinkers and their influences, and group them into seven schools of thought. Two major trends can be distinguished. One is the associationist trend, starting with the work of Locke and Hume, developed by Hartley, Bain, and Mill to be later absorbed into behaviorism through pioneering animal psychologists (Morgan and Thorndike). This tradition conceived of habits atomistically and as automatisms (a conception later debunked by cognitivism). Another historical trend we have called organicism inherits the legacy of Aristotle and develops along German idealism, French spiritualism, pragmatism, and phenomenology. It feeds into the work of continental psychologists in the early 20th century, influencing important figures such as Merleau-Ponty, Piaget, and Gibson. But it has not yet been taken up by mainstream cognitive neuroscience and psychology. Habits, in this tradition, are seen as ecological, self-organizing structures that relate to a web of predispositions and plastic dependencies both in the agent and in the environment. In addition, they are not conceptualized in opposition to rational, volitional processes, but as transversing a continuum from reflective to embodied intentionality. These are properties that make habit a particularly attractive idea for embodied, enactive perspectives, which can now re-evaluate it in light of dynamical systems theory and complexity research.

## Introduction

**Figure 1:**
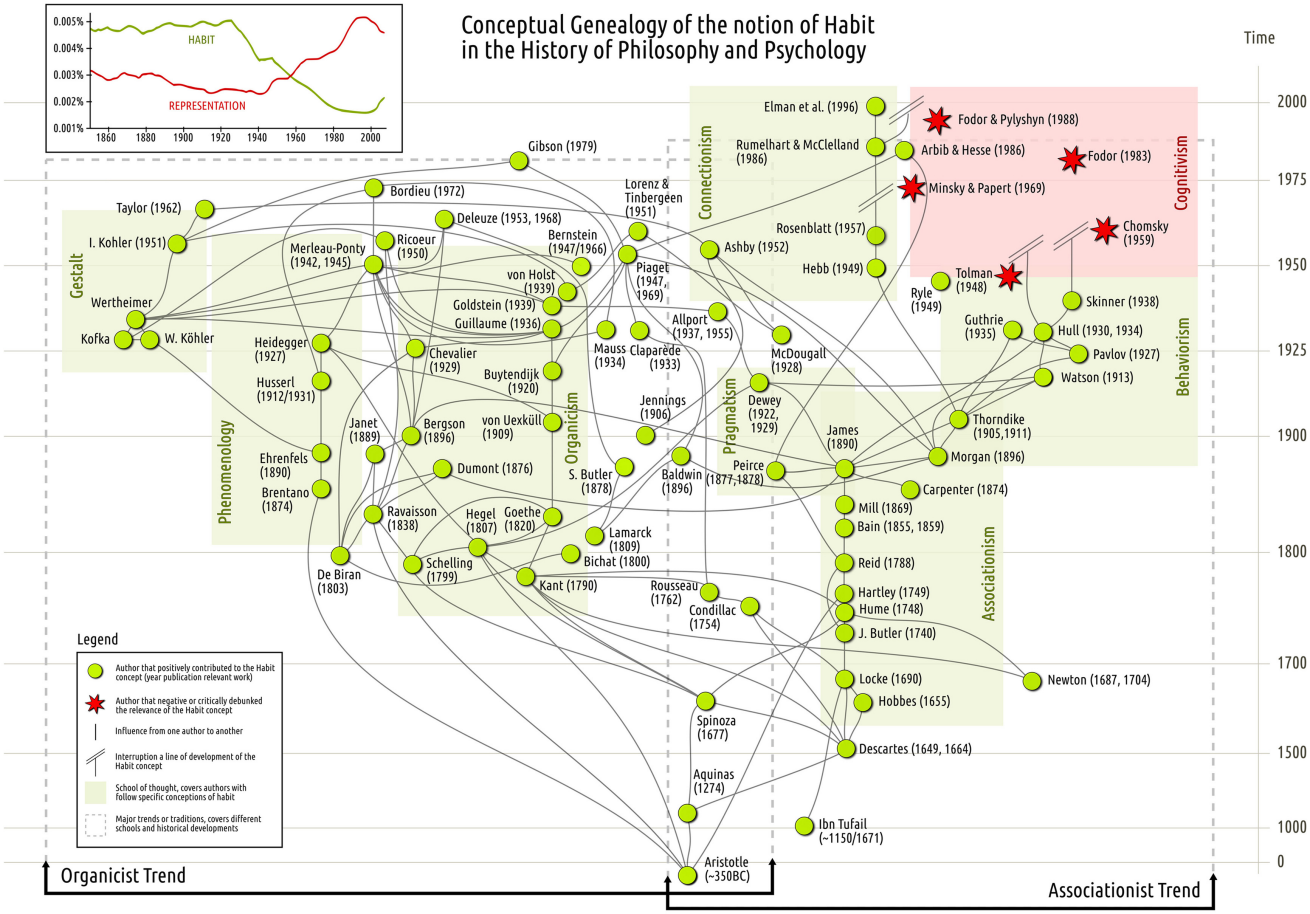
**Conceptual genealogies of the habit concept.** The plot at top-left corner of the map displays the ngram of the terms “habit” and “representation” in non-fiction literature (in English) published between 1850 and 2008 and scanned by Google, see Michel et al. (2011) for more details. Green circles indicate positive contributors to the concept of habit. Red stars indicate breaks in the development or significance of the habit concept. A high definition version of the map can be found here: http://barandiaran.net/design/habit-map.

For over 60 years the most basic theoretical concept in psychology, neuroscience, and cognitive science has been the processing of information and the associated notion of “mental representation.” Neuroscientists search for modules and regions that process, store, retrieve or integrate information that is encoded or represented in the brain. But this hasn't always been the case. Before the advent of cognitivism in the 1950s one of the most prominent concepts for the study of mind was that of *Habit*. Despite constituting only very coarse evidence, the sub-plot in Figure 1 (top-left) shows trends in the use of the words “habit” and “representation” since 1850. It is noteworthy that for most of the second half of the 20th century mentions of “habit” decrease and those of “representation” increase in a sustained manner. The anti-correlation is maintained with the reversal of these tendencies at the start of the 21st century, roughly indicating that habit is again becoming a notion of interest. This is no coincidence. Current embodied dissatisfactions with the information-processing framework (Varela et al., 1991; Kelso, 1995; Van Gelder, 1998; Thompson, 2007; Chemero, 2009; Di Paolo et al., 2010; Hutto and Myin, 2013) call for a reappraisal of this notion. The task, one quickly finds, is huge. The richness and polysemy of the notion of habit and its transformations since ancient Greece to the present day, all militate against the naïve idea of producing an off-the-shelf alternative theoretical primitive for psychology and neuroscience.

In this mini-review we offer a brief genealogy of the concept of habit in the form of a network-map. We place those thinkers who have worked on this concept in a historical relation. Our objective is to outline the genealogy of the notion of habit and identify major trends and schools of thought that have had an impact on current neuroscientific conceptions of habit and those that have not but still deserve attention.

As in the case with real maps, there is potentially no end to the amount of detail that may be included. The more detailed the map, the better the chances for efficient local navigation, but often at the price of losing the big picture. We have chosen to draw only the big picture (Figure 1). For this reason, links represent a general notion of “influence” between two thinkers, without going into relevant details such as, e.g., whether the influence has been positive or critical, whether it is manifested as an explicit conceptual debt or as more subtle forms of inspiration, or indeed whether the same thinker's notion of habit has evolved significantly at different stages and under different influences.

It is likely that no two links in our map depict the exact same kind of influence. But a link describes at least an acknowledged or clearly recognized impact, which in most cases will be manifested as a direct reference to the influencing thinker in the works listed on Table 1. As a general rule transitive influences have not been drawn on the map and antagonistic links are also left out unless the critique of a previous conception of habit leads to a richer conception that integrates the view of the criticized author.

**Table 1:** **List of authors and their most significant work related to habits. The year corresponds to the original publication and the title to the English translation (if available)**.

**Year**	**Author**	**Work**
−350	Aristotle	Nichomachean ethics, Metaphysics, De anima, De memoria and, Categories
~1150/1671	Ibn Tufail	Philosophus autodidactus [Risala Hayy ibn Yaqzan fi asrar al-hikmat al-mashriqiyya]
1274	T. Aquinas	Summa theologica (Treatise on habit QQ49-54)
1649, 1664	R. Descartes	The passions of the soul and Treatise of man
1655	T. Hobbes	De corpore
1677	B. Spinoza	Ethics
1687, 1704	I. Newton	Philosophiae naturalis Principia mathematica and Opticks
1690	J. Locke	An essay concerning human understanding
1739, 1748	D. Hume	A treatise of human nature and Enquiry concerning human understanding
1740	J. Butler	The analogy of religion, natural and revealed, to the constitution and course of nature
1749	D. Hartley	Observations on man, his frame, his duty, and his expectations
1754	E. B. de Condillac	Treatise on the sensations
1762	J-J Rousseau	Émile or On education
1788	T. Reid	Essays on the active powers of the human mind
1790	E. Kant	Critique of judgment
1799	F. W. J. Schelling	First outline for a system of a philosophy of nature
1800	X. Bichat	Recherches physiologiques sur la vie et la mort
1803	M. de Biran	Influence de l'habitude sur la faculté de penser
1809	J. B. P. Lamarck	Zoological philosophy
1820	J. W. Goethe	Outline for a general introduction comparative anatomy, Commencing osteology
1830	G. W. F. Hegel	The philosophy of mind (Part 3 of the Encyclopaedia of the philosophical sciences)
1838	F. Ravaisson	Of habit
1855, 1859	A. Bain	Senses and the intellect, The emotions and the will
1869	J. Mill	Analysis of the phenomena of the human mind
1874	W. B. Carpenter	Principles of mental physiology
1874	F. C. Brentano	Psychology from an empirical standpoint
1876	L. Dumont	De l'habitude (Rev. Phil de la France et de l'Etranger)
1877, 1878	C. S. Peirce	The fixation of belief, How to make ideas clear (see also Collected Papers)
1878	S. Butler	Life and habit
1889	P. Janet	L'Automatisme psychologique
1890	C. von Ehrenfels	Über Gestaltqualitäten
1890	W. James	Principles of psychology (Ch. 4 Habit)
1896	J. M. Baldwin	Mental development in the child and the race: Methods and processes
1896	C. L. Morgan	Habit and instinct
1896	H. Bergson	Matter and memory
1905, 1911	E. Thorndike	Elements of psychology, Animal intelligence: Experimental studies
1906	H. S. Jennings	Behavior of the lower organisms
1909	J. von Uexküll	Umwelt und Innenwelt der Tiere
1912	E. Husserl	Ideas: General introduction to pure phenomenology (Part II)
1913	J. B. Watson	Psychology as the behaviorist views it
1920	F. J. J. Buytendijk	Psychologie der dieren
1922, 1929	J. Dewey	Human nature and conduct, Experience and Nature
1927	M. Heidegger	Being and time
1927	I. P. Pavlov	Conditioned reflexes
1928	W. McDougall	Body and mind; A history and a defence of animism
1929	J. Chevallier	L'habitude: essai de métaphysique scientifique
1930, 1934	C. L. Hull	Knowledge and purpose as habit mechanisms, The concept of the habit-family hierarchy and maze learning
1933	E. Claparède	La Genèse de l'hypothèse: étude expérimentale
1934	M. Mauss	Techniques of the body
1934	K. Goldstein	The organism
1935	E. von Holst	Relative coordination as a phenomenon and as a method of analysis of central nervous system function
1935	E. R. Guthrie	The psychology of learning
1936	P. Guillaume	La formation des habitudes
1937, 1955	G. Allport	The functional autonomy of motives, Becoming
1938	B. F. Skinner	The behavior of organisms: An experimental analysis
1942, 1945	M. Merleau-Ponty	The structure of behavior, Phenomenology of perception
1947, 1969	J. Piaget	The psychology of intelligence, Biology and knowledge
1947/1967	N. Bernstein	The co-ordination and regulation of movements see also Dexterity and its development (1996)
1948	E. C. Tolman	Cognitive maps in rats and men
1949	G. Ryle	The concept of mind
1949	D. Hebb	Organization of behavior
1950	P. Ricoeur	Freedom and nature: The voluntary and the involuntary
1951	K. Lorenz & N. Timbergeen	The study of instinct
1951	I. Kohler	The formation and transformation of the perceptual world (1964)
1952	W. R. Ashby	Design for a brain
1953, 1968	G. Deleuze	Difference and repetition, Empiricism and subjectivity
1957	F. Rosenblatt	The perceptron: A probabilistic model for information storage and organization in the brain
1959	N. Chomsky	A review of B. F. Skinner's Verbal behavior
1962	J. G. Taylor	The behavioral basis of perception
1969	M. L. Minsky & S. Papert	Perceptrons: An introduction to computational geometry
1972	P. Bourdieu	Outline of a theory of practice (1977)
1979	J. J. Gibson	The ecological approach to visual perception
1983	J. Fodor	The modularity of mind: an essay on faculty psychology
1986	M. A. Arbib and M. B. Hesse	The construction of reality
1986	D. E. Rumelhart, J. L. McClelland and PDP Group	Parallel distributed processing, Vol. 1: Foundations
1988	J. Fodor and Z. W. Pylyshyn	Connectionism and cognitive architecture: A critical analysis
1996	J. L. Elman et al.	Rethinking innateness: A connectionist perspective on development

We have taken the general rule that all authors presented on the map should have discussed habits explicitly. But there are a few exceptions to this rule. For instance, Kant did not elaborate a strong positive contribution to the notion of habit—in fact, he is accountable for the ensuing divide between habit and reason in ethics—yet, his insights into the nature of teleology and self-organization strongly influenced the notion of habit, plasticity and holistic interdependence in various thinkers. Others do not make direct use of the term habit, but use parallel notions that were later (or previously) conceptualized as habits (such as von Uexküll's “functional cycles” or Pavlov's “reflexes”). The map still leaves out a considerable amount of literature on habit or habit-related research, e.g., work in economics, anthropology, psychoanalysis and research on habituation and addiction.

The timeline reaches up to the 1980s with some additional references to later work in the cognitivist and connectionist traditions added for completeness (Elman, Rumelhart, and McClelland, Arbib, Fodor, etc.). It is worth noting that a few authors appear (almost) without connections (von Holst, Bernstein, Ryle), yet their contributions are nowadays considered important. Gestalt psychologists, who together exert a notable influence on the habit concept without addressing it directly in their work, appear without a reference in Table 1.

The reader might still be left with a fundamental question regarding the key contribution of this map: What is the value of this genealogy for contemporary neuroscience? Whereas much work in human neuroscience appears informed by a rich philosophical, psychological and theoretical tradition (e.g., the neuroscience of perception, emotion or consciousness, cognitive or large-scale neuroscience), we believe that neuroscientific research on habit remains rooted within a narrow theoretical tradition. For instance, in an otherwise excellent review of recent work, Graybiel (2008) makes only a sparse reference to William James. Similarly, Wood and Neal (2007) only mention Thorndike and Skinner as conceptual precursors. This is understandable, as the history of habit is indeed complex and relatively unexplored. Our inherited conception appears historically distorted—only a few recent studies examine the genealogy of the concept (see Pollard, 2008; Carlisle and Sinclair, 2011; Carlisle, 2014). The map we present is an attempt to fill in this gap, providing a birds-eye view that can be used to navigate the history of the concept.

## Tracing the genealogies of habit

Let us attempt a broad reading of the map. We identify two major historical trends, associationism and organicism, taking their names from the most salient school of thought in each trend. But we shall first start from the Greek and Aristotelian polysemic conception of habit.

The Latin term *habitus*, from which the English *habit* comes, can be traced back to two Greek words: *ethos* (ἔθ*o*ς), and *hexis* (ἕξις). The etymology of *ethos*, from which the English term *ethics* derives, is particularly revealing because it contains a profound duality. It means both “an accustomed place” in which human and animals live or in-habit (a “habitat”) and “a disposition or character” denoting the personality that develops along a person's lifetime. According to Aristotle, the term *hexis* (having or being in possession of something) is a relational and active category: “a kind of activity of the haver and of what he has—something like an action or movement” [Met. 5.1022b]^1^, it is also a normative dispositional category “‘Having’ or ‘habit’ means a disposition according to which that which is disposed is either well or ill disposed” [Met. 5.1022b]. The ethical implications of this conception of habit extend to a self-modifying practice, exercised so as to attain a virtuous character wherein spontaneity, joy, and norms converge.

We can interpret the Aristotelian conception of habit as an arrangement of behavioral mediations between subject and object (or between a subject and herself—in the future or past) that is well or ill-disposed in relation to essence or form and the “immediate substrate in which it is naturally produced.” Habit arises from custom or repetition in a manner that constitutes a sort of second nature for the subject. In this sense, Aristotle can be said to be one of the early precursors of the organicist trend in the conception of habit. But he is also credited for inspiring the central claim of associationism (Buckingham and Finger, 1997).

### The associationist trend

Associationism can be summarized as the view that mental phenomena are formed by combination or association of simple elements. This association follows the principle that the occurrence of event B given event A will be favored if B has repeatedly followed A in the past (often, the strength of A or B, their similarity, space-time contiguity, etc. are taken as strengthening this association). A and B are generally considered as mental states or ideas arising from sensations (often interpreted in terms of nervous activation).

The work of Ibn Tufail (12th century), translated into Latin as *Philosophus Autodidactus* [1671], tells the story of a child that reconstructs a full philosophical and theological system without the help of a social or cultural environment. It influenced the first associationists, particularly John Locke whose notion of *tabula rasa* was almost directly taken from Ibn Tufail (Russell, 1994). Locke's empiricist principle—that sense data had to fill in a blank slate—provided the basis of what was to come although he didn't provide a detailed account of associationism^2^.

It was David Hume [1748] who proposed the notion of “habit,” “custom,” or “association” as the fundamental mechanism for the development of psychological and epistemological complexes. Atomized ideas are the direct result of sensations, while the law of habit becomes the general principle of mental organization by linking these ideas. Newton's influence on this conception of habit is apparent. Although the principles established by Hume are not fundamentally modified by Hartley's work, the latter was capable of extending them to many psychological phenomena (from memory to language, psychological development and emotions). Perhaps one of the most salient contributions was Hartley's account of habits as arising from “corporeal matter,” completing Hume's philosophical approach with an influential neuro-physiological theory of associations based on the operations of the brain and the spinal cord, in accordance with the “doctrine of vibrations” previously suggested by Newton (Glassman and Buckingham, 2007). Further contributors to the associationist school (Bain, Mill, Carpenter, etc.) conserved most of the principles and theoretical assumptions of Hume and Hartley until a scientific formulation of some of these principles by behaviorist precursors came from the scientific study of animal behavior (Morgan, Thorndike and Pavlov).

The subsequent development of the notion of habit was subordinated to the available methods of measurement and intervention, which aimed at the “prediction and control of behavior” [Watson, 1913: 158]. The contribution of behaviorism to this trend can be summarized in two main aspects that result from the epistemological constraints of logical positivism (Smith, 1986) on the notion of habit: (a) the progressive externalization of the units of association in terms of stimulus and response (removing any reference to intermediate neurological or psychological processes) and (b) the mathematical treatment of the relationship between external operators and observables (stimulus, response, reinforcers) in terms of conditional probabilities. Skinner even rejected learning theories (Skinner, 1950) and purified the available terminology dropping the notion of habit altogether in favor of “rate of conditioned response.”

At this point, together with the advent of computational and information theory, the ground was prepared for the now much impoverished notion of habit to disappear altogether from the set of theoretical primitives in psychology and neuroscience. Through experimental [Tolman, 1948] and theoretical [Chomsky, 1959; Fodor, 1983] arguments against behaviorism, habit was soon replaced by “mental representation” and the notion of “association” was substituted by that of “computation.” Some of the associationist (and also organicist) principles were revived in neuroscience [Hebb, 1949] and, particularly, in connectionism [Rosenblatt, 1957; Rumerhart and McClelland, 1987] only to be fiercely attacked again by cognitivists [Minsky and Papert, 1969; Fodor and Pylyshyn, 1988]^3^. The result of this development is the current convergence of machine learning and reinforcement learning with neuroscience (see Sutton and Barto, 1998; Daw et al., 2005; Dezfouli et al., 2012) where habits have been subsumed under networks of conditional probabilities of expected rewards associated with a set of available actions under specific conditions, or simply reduced to stimulus-triggered responses reinforced only by repetition (Dickinson, 1985). Associationist principles still exert an influence in neuroscience under the form of Hebbian learning and activity-dependent plasticity (Abbott and Nelson, 2000).

### The organicist trend

Somewhat parallel to the development of the associationist trend we encounter an organicist tradition (left of Figure 1). Habits in this tradition are examined along what we would call today more ecological, self-organizing lines. Habits are both cause and effect of their own enactment and therefore constitute their own principle of individuation (Toscano, 2006), as opposed to being the passive result of the recurrence of an otherwise pre-established set of entities (ideas, stimulus, rewards, etc.). For organicism, habits are also related to a plastic equilibrium that involves the totality of the organism, including other habits, the body and the habitat they co-determine.

Spinoza's notion of *conatus*, as the striving for perseverance that defines the essence of organisms, prefigured the internalist and naturalistic conception of individuality and teleology that characterizes organicism. Kant [1790] provided a regulative notion of teleology in terms of the intertwinement of means and ends in the self-organized nature of organic life, thereby insinuating a way out of the tight mechanistic framework established by Descartes and Newton. Hegel [1830], in deep dialog with the Aristotelian tradition, emphasized the plasticity of habit as the mediating term in the resolution of the mind's contradictory tendencies toward world-independence and self-determination on the one hand, and over-stimulation and world-determination, on the other. By becoming second nature, habit prevents the mind from falling into either extreme that would lead to insanity. Goethe (though not directly addressing the notion of habit) deeply influenced subsequent conceptions of organic life by coining the term “morphology” and proposing the *law of compensation* to refer to the plastic change of natural forms in accordance with inner forces that respect the balance of the totality [1820]. Ravaisson's *De l'habitude* [1838] constitutes a cornerstone within this trend. Ravaisson puts habits at the center of metaphysics, extending from vegetative life to deliberative thought, defining habits as dynamical processes that transverse a continuum between reflective/self-aware and pre-reflective/embodied forms of intentionality (Sinclair, 2011).

Further development of the habit notion within the organicist school made it possible to expand on the dialectics between the inner tendencies of organic individuality and its co-development with the environment. von Uexküll [1909] used the term *Umwelt* to designate the *habitat* of the organism, that is, the carving of a world (from an undifferentiated environment) through functional sensorimotor cycles. His work was part of an organicist revival in Central Europe during the first half of the 20th century (Grene, 1965; Harrington, 1996), with notable exponents like the phenomenologically-informed psychologist/ethologist F. J. J. Buytendijk and neurologist Kurt Goldstein, whose studies of abstract vs. concrete behaviors in patients with brain lesions led him to holistic notions of the organism as seeking the equilibrium of preferred behaviors.

Somewhat intertwined within the organicist school, phenomenology and Gestalt psychology enriched this tradition in various ways. Husserl, for instance, acknowledged that habit is “intimately involved in the constitution of *meaningfulness*” at all levels, from perception to society (Moran, 2011). Merleau-Ponty [1945] drew inspiration from Paul Guillaume's Gestalt approach and Goldstein's experiments to develop a notion of habits as incorporated styles of being-in-the-world, thus revealing their inherent corporeal intentionality in contrast to notions of habits as blind automatisms. Gestalt psychology provided a systematic and experimental basis for holistic phenomena in perception, displacing atomistic metaphors in psychology in favor of fields and systems theory. Of particular significance are the experiments with vision distorting goggles by Ivo Kohler [1962] who emphasized the importance of action for perception, combining an active notion of habit with Gestalt principles.

Overall, the exponents of the organicist tradition in the 20th century pronounced themselves explicitly against atomistic tendencies, such as the localization of brain function and theories of reflex conditioning. The trend also influenced pragmatist thinkers such as James [1890], and particularly Dewey [1922], who also saw habits as communicating wholes affecting each other and as the substrate of self-transforming human nature. He resisted the reductionist implications of the reflex-arc concept by highlighting the active role of the organism in the selection of stimuli.

Organicism, whose ramifications appear less unified and cumulative than the associationist line, has influenced a variety of positions ranging from the integrative work of Piaget (his treatment of habit marks the starting point for a dynamic conception of cognitive development) to ecological psychology [Gibson, 1979], and the sociological conception of *habitus* as structured and structuring practices [Mauss, 1934; Bourdieu, 1972].

Current sensitivity to the organicist trend is manifest in large-scale neuroscience (Edelman and Tononi, 2001; Freeman, 2001; Llinas, 2001), constructivist developmental neuroscience (Quartz and Sejnowski, 1997; Johnson, 2001), embodied-enactive cognitive science (Varela et al., 1991; Thompson, 2007; Di Paolo et al., 2010), robotics (Di Paolo, 2003; Egbert and Barandiaran, under review), sensorimotor approaches to cognition (O'Regan and Noë, 2001; Noë, 2004) and cognitive neuroscience (Engel et al., 2013). In most cases the concept of habit forged by the organicist tradition has been modified to avoid the critiques against behaviorism, and its legacy appears to be masked under related notions such as skill, sensorimotor organization, neuroplasticity, etc.

## Conclusions

We have provided a map and a very broad survey of the various ways in which the concept of habit has evolved from ancient Greece to the late 1980s, identifying two major traditions. The associationist trend conceives of habits atomistically as units that result from the association of ideas or between stimulus and response. The organicist trend, in contrast, sees habits as dynamically configured stable patterns, strengthened and individualized by their enactment. Associationism provides a statistical or combinatorial relationship between the components of a habit (based on time lapses between events, their similarity, etc.). Organicism, in contrast, proposes a more holistic view, wherein embodied relational constraints and plastic interdependencies determine the formation and maintenance of habits. Finally, the associationist trend keeps habit within the realm of reactive sub-personal automatisms (in opposition to the intentional, rational, and personal levels of cognitive processing). For organicism, in contrast, habits transition between nature and will, forming an integral part of individual embodied intentionality; they are the systemic conditions of the possibility of experience—their significance becomes clearly manifested when habits are disturbed yet they remain continuously present, configuring the identity and world of the cognitive subject.

Unlike many notions in organicism, associationist ideas were ready-made for translation into scientific hypotheses during the 20th century, even if it was ironically the subsequent development of such formalisms that fueled the cognitivist rejection of the notion of habit. While neuroscience has been partially influenced by this rejection, related ideas have survived, particularly in theories of neuroplasticity and Hebbian learning. These habit-like notions are generally associationist in character, but they have also given rise to theories of neural assemblies and neural self-organization (Varela, 1995; Freeman, 2001), which are more organicist-friendly. Similarly, in other areas of cognitive science, dynamical systems formalisms, modeling and experimental techniques now provide the necessary tools for investigating more organicist conceptions of habit.

### Conflict of interest statement

The authors declare that the research was conducted in the absence of any commercial or financial relationships that could be construed as a potential conflict of interest.

## Footnotes

^1^References given between square brackets correspond to works used in constructing the map. They are listed in Table 1.

^2^Except for chapter 33 in his Essay introduced only in the 4th edition and dealing mostly with the origin of confusion and mistaken ideas.

^3^For a detailed account of these developments and intellectual battles, see Margaret Boden's monumental history of cognitive science (Boden, 2006).
